# Target-Dependent Enrichment of Virions Determines the Reduction of High-Throughput Sequencing in Virus Discovery

**DOI:** 10.1371/journal.pone.0122636

**Published:** 2015-04-08

**Authors:** Randi Holm Jensen, Sarah Mollerup, Tobias Mourier, Thomas Arn Hansen, Helena Fridholm, Lars Peter Nielsen, Eske Willerslev, Anders Johannes Hansen, Lasse Vinner

**Affiliations:** 1 Centre for GeoGenetics, Natural History Museum of Denmark, University of Copenhagen, Copenhagen, Denmark; 2 Department of Epidemiology Research, Statens Serum Institut, Copenhagen, Denmark; University of North Texas Health Science Center, UNITED STATES

## Abstract

Viral infections cause many different diseases stemming both from well-characterized viral pathogens but also from emerging viruses, and the search for novel viruses continues to be of great importance. High-throughput sequencing is an important technology for this purpose. However, viral nucleic acids often constitute a minute proportion of the total genetic material in a sample from infected tissue. Techniques to enrich viral targets in high-throughput sequencing have been reported, but the sensitivity of such methods is not well established. This study compares different library preparation techniques targeting both DNA and RNA with and without virion enrichment. By optimizing the selection of intact virus particles, both by physical and enzymatic approaches, we assessed the effectiveness of the specific enrichment of viral sequences as compared to non-enriched sample preparations by selectively looking for and counting read sequences obtained from shotgun sequencing. Using shotgun sequencing of total DNA or RNA, viral targets were detected at concentrations corresponding to the predicted level, providing a foundation for estimating the effectiveness of virion enrichment. Virion enrichment typically produced a 1000-fold increase in the proportion of DNA virus sequences. For RNA virions the gain was less pronounced with a maximum 13-fold increase. This enrichment varied between the different sample concentrations, with no clear trend. Despite that less sequencing was required to identify target sequences, it was not evident from our data that a lower detection level was achieved by virion enrichment compared to shotgun sequencing.

## Introduction

Viral infections continue to be an important cause of diseases [1, 2]. Recently, novel viruses such as influenza A H7N9 variants [3, 4] and Middle East Respiratory Syndrome coronavirus (MERS-CoV) [5, 6] have been discovered. Both of these cases exemplify the importance of continued surveillance for new pathogens, due to the risk of outbreaks or epidemics. Furthermore, viral infections are believed to cause approximately 15–20% of human cancers [7]. The known oncogenic viruses belong to highly divergent virus genera and include human papillomaviruses (HPVs), hepatitis B and C virus, Epstein-Barr virus, Merkel cell polyomavirus, human T cell-lymphotropic virus type-1 and Kaposi’s sarcoma herpesvirus [8, 9]. As the etiology of many cancers is still unknown, it is not unlikely that yet unidentified oncogenic viruses exist that infect humans, and it is therefore important that methods for the discovery of novel viruses are continually developed and improved.

The diversity of viral families is tremendous, both in morphology, genome size, and in the genomic organisation that may be single or double-stranded DNA or RNA in a linear or circular conformation. In contrast to bacterial ribosomal DNA [10], no common genetic marker exists among viral genomes that ensure detection of all genetic variants including novel genera or species [11, 12]. Traditionally, viruses have been discovered using immunochemical methods, electron microscopy and cell culture. Molecular methods such as PCR and microarrays have been employed more recently [13–16]. However, most sensitive molecular methods are also highly specific, and require prior knowledge of the target sequence. The lack of a common viral genetic marker makes viral discovery difficult, and even the detection of novel subtypes can be challenging [11, 12].

High-throughput sequencing (HTS) requires no prior knowledge of the target sequences. In theory, unbiased HTS investigation should increase the probability of identifying novel viruses in various diseases, such as cancers and in infections, when conventional tests fail.

Numerous methods are currently available for targeting genomic material using HTS. One simple approach is shotgun sequencing where sequencing libraries are prepared from all available sample DNA and/or RNA present in a sample. Viral sequences typically represent a very limited fraction compared to host-derived sequences, constituting a significant level of irrelevant background data. Furthermore, the genome size of viruses is also significantly smaller than the host genome. Consequently, extensive shotgun sequencing may be necessary for detection of viral targets.

Alternatively, methods for target enrichment should be considered for detection of viral sequences in samples with low or unknown concentrations of virus. Virion nucleic acid enrichment by physical removal of host material may be combined with subsequent specific amplification of target nucleic acids either before or after preparation of the sequencing libraries [14, 17–19]. Virion enrichment utilizes the characteristic that viral genomes are protected by protein capsids, and for some viruses also a lipid envelope. Centrifugation of homogenized samples followed by filtration removes host cells and cellular debris. Leftover unprotected host nucleic acids can be removed with nucleases, theoretically leaving only enriched encapsidated viral DNA and RNA for extraction [20, 21]. Furthermore, target enrichment may be obtained by hybridization of specific probes to the viral target DNA/RNA [15, 22] or capture of host material using specific probes for subtractive hybridisation [23]. However, the use of such methods requires some knowledge to the sequences of the target and can thus be biased.

The implementation of HTS techniques together with continually decreasing costs [24] has provided new possibilities of discovering pathogens. An example is the discovery of a novel arenavirus in samples where conventional PCR, cell culture and serological assays had previously failed to detect pathogens [25]. However, the sensitivity of HTS techniques included in studies identifying novel viruses is most often not established [17, 19, 26], and may well vary depending on the target virus, sample type and enrichment technique [27]. Hence, major challenges still exist, as the sensitivity and the enrichment efficiency of the different HTS techniques have not been thoroughly examined.

**Fig 1:**
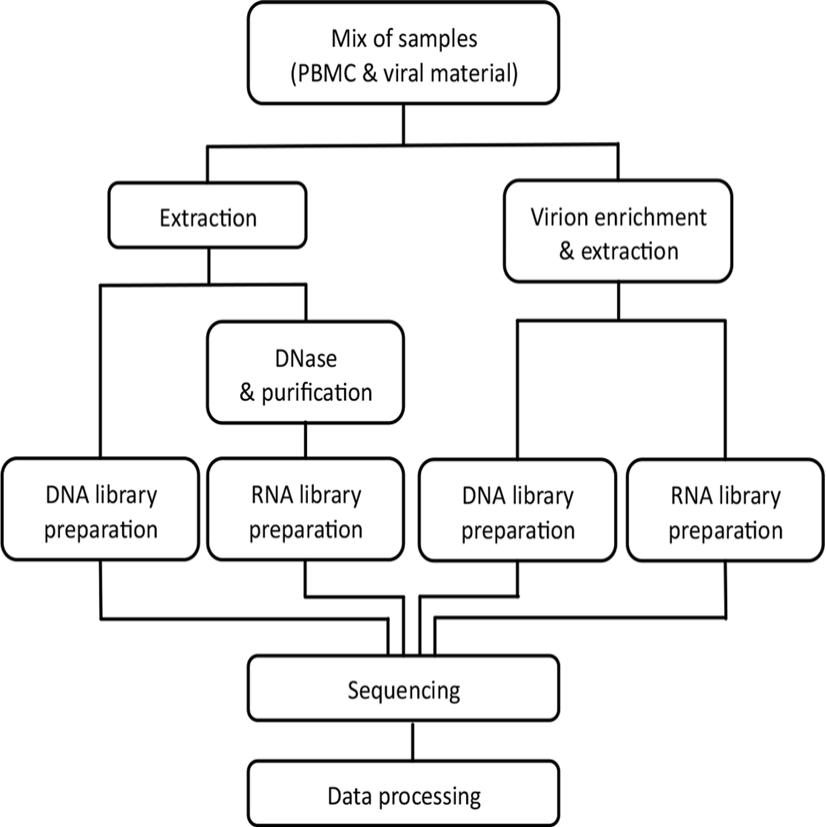
Overview of the experimental design. Fractions of each sample, consisting of varying viral material spiked into human PBMCs were subjected to shotgun sequencing of DNA or RNA. On other fractions virion enrichment procedures including centrifugation, filtration and nuclease treatment were performed prior to library preparation (DNA or RNA) and sequencing.

This study aimed at determining the sensitivity of different HTS library preparation procedures commonly used for viral discovery. For decreasing concentrations of target, we compared the effect of virion enrichment. To mimic the complexity of a biological sample, test sample material was generated containing different types of virions and/or infected human cells, spiked to a pool of human peripheral blood mononuclear cells (PBMCs). To represent some of the diversity between viral families, we included viruses with either DNA or RNA genomes, non-enveloped viruses, proviruses integrated into the human genome in single or multiple copies per genome, plasmid DNA, as well as armored RNA (aRNA). An important aspect of this investigation was to determine the level of enrichment compared to the need of sequencing depth in shotgun sequencing. Each sample was split in four fractions of which two were used for shotgun sequencing of total DNA or RNA. The remaining two fractions were subjected to virion enrichment, and libraries were prepared on virion-associated DNA or RNA, from here on referred to as virion-enriched libraries (Fig 1).

## Methods

### Ethics statement

Human PBMCs were obtained from an anonymous blood donor from the blood bank at Copenhagen University Hospital (Rigshospitalet). The present study was performed within a larger frame program, which was submitted for ethical evaluation to the Regional Committee on Health Research Ethics and the National Committee on Health Research Ethics. As the included human cells are completely anonymous, both ethical boards waived the need for ethical permission, and consequently informed consent (case no. H-2-2012-FSP2 and 1304226, respectively) according to Danish national legislation (Sundhedsloven).

### Sample material

Seven different test samples were prepared by spiking PBMCs from anonymous blood donors with known concentrations of different viral material (Table 1). The material used was HeLa cells harbouring 10–50 copies of integrated human papilloma virus type 18 (HPV-18), 8E5 cells carrying one copy of proviral human immunodeficiency virus 1 (HIV-1) genome, enterovirus B Coxsackievirus B3 virions (EV), human adenovirus C virions (HAdV), a plasmid encoding a copy of measles virus [28] (MeV plasmid), and armoured RNA (aRNA) carrying a 263bp fragment of the 5’UTR of an enterovirus genome (Asuragen, Austin, TX, USA). The range in copy number of the different viruses in the samples were based on the sensitivity of the specific quantitative PCR (qPCR) assays and thus varied among the different virus types, with the lowest concentration aimed at the detection limit of the qPCR assays. The accumulated number of cells was equal in each sample (PBMCs, HeLa and 8E5 cells). Each sample was divided into four fractions and different laboratory procedures were applied to target either the total DNA or RNA content or the virion-associated DNA or RNA.

**Table 1:** Quantity of virus in complex control sample material.

	**Copy number of cells or virus per μl sample**					
**Sample**	**HIV-1, provirus**	**HPV-18, integrated**	**HAdV**	**EV**	**MeV plasmid**	**aRNA**
1	4,000	0	0	0	700	5
2	400	0.4	2.5	12.5	700	5
3	40	0.4	2.5	12.5	70	5
4	4	4	25	125	70	5
5	0.4	40	25	125	7	5
6	0.4	400	250	1,250	7	5
7	0	4,000	2,500	12,500	0	5

HIV-1; Human Immunodeficiency Virus type-1, HPV-18; Human papillomavirus type 18, HAdV; Human Adenovirus C, EV; Enterovirus B Coxsackievirus B3, MeV; Measles virus, aRNA; armored RNA.

### Cell cultures

Human HeLa cells containing 10–50 proviral copies/cell of HPV-18 [29, 30] were grown in E-MEM (Sigma-Aldrich) containing 10% inactivated fetal bovine serum (FBS), 120 IU penicillin/ml, 120 μg streptomycin/ml, and 2mM L-Glutamin. Human 8E5 cells containing a single copy/cell of proviral HIV-1 [31, 32] were grown in RPMI-1640 with Glutamax-I (Invitrogen) containing 120 IU/ml penicillin, 120 μg/ml streptomycin, 24 μg/ml gentamycin, 6 IU/ml nystatin, and 10% inactivated FBS.

### Reverse transcription and qPCR

Reverse transcription (RT) was performed for the EV and aRNA on 2 μl extract, using random hexamers and SuperScript III (Invitrogen) according to manufacturer’s instructions. The concentrations of the different viruses in the samples were determined by qPCR using the Lightcycler 480 Probes Master mix reagents (Roche) including 500 nM target specific primers and 200nM probes (Table 2), 2 μl of template and H_2_O to a final volume of 20 μl.

**Table 2:** PCR primer pairs and probes used for amplification.

**Target**	**Product size**	**Sequence (5’-3’) incl. dyes and quenchers**
HIV-1^#^	128 bp	AAGCCTCAATAAAGCTTGCCTTGA
		GTTCGGGCGCCACTGCTAG
		FAM-TCTGGTAACTAGAGATCCCTCAGACC-BHQ1
HPV-18	142 bp	TCACGAGCAATTAAGCGACTCA
		TAGCTCAATTCTGGCTTCACACTT
		FAM-AATCATCAACATTTACCAGCCCGACGAG-BHQ1
HAdV	132 bp	CAAATCCCACTGTCACATGCA
		GCCACGGTGGGGTTTCTAAACTT
		FAM-TGGTTGAGTTGGACCCGATAA-BHQ1
EV or aRNA	143 bp	TGAATGCGGCTAATCCCAAC
		ATTGTCACCATAAGCAGCCA
		FAM-AACCGACTACTTTGGGTGTCCGT-BHQ1
MeV plasmid	94 bp	TTATGTGTTCTGGCAGATTTGTACTG
		GGTCGGTTAGAACTGCACATTTC
		FAM-CAACCAGGGACCTGCCCACCA-BHQ1

^#^ Drosten et al. [33].

For the HPV-18, EV and aRNA, MeV plasmid, and HAdV-C assays the qPCRs were performed by denaturation at 95°C for 10 min followed by 40 amplification cycles with denaturation at 95°C for 10 sec and annealing, elongation, and real time fluorescence measurement at 60°C (55°C for the adenovirus assay) for 1 min. For the HIV-1 assay the PCR run was performed according to Drosten et al. [33].

The qPCR standards consisted of counted 8E5 cells or HeLa cells, an adenovirus control of 100,000 copies/ml (AcroMetrix), aRNA with a concentration of 50,000 copies of EV/ml (Asuragen), and for the MeV plasmid the molar concentrations was calculated from the plasmid size. Standard curves were generated from triplicates of serially diluted standards for each qPCR run to determine the concentration of virus in the samples. All viruses could be detected by qPCR down to the lowest concentration used in the prepared samples.

### DNA and RNA preparations

Extracts of total nucleic acid were obtained by using the QIAamp DNA mini kit (Qiagen) according to manufacturer’s instructions with the addition of 10 μg linear acrylamide as carrier (Applied Biosystems) at the ethanol precipitation step. QIAamp DNA mini kit was used for both DNA and RNA extractions, as RNA yields equalled that of commonly used RNA columns (RNeasy mini kit). Virion enrichment was performed by centrifugation for 2 minutes at 800 × g to remove tissue debris, and the supernatants were subsequently filtered through 5 μm centrifuge filters (Millipore). In addition to virus discovery, the methods were also designed for inclusion of bacteria, and thus a filter pore size of 5μm was chosen for the filtration. The filtrates were nuclease treated to remove unprotected nucleic acids using 7 μl TURBO DNase (2U/μl) (Ambion), 6 μl Baseline-ZERO DNase (1U/μl) (Epicentre), 8 μl RNase Cocktail Enzyme Mix (Ambion), and 20 μl 10× TURBO DNAse buffer in a final volume of 200 μl, and incubated at 37°C for two hours. Viral nucleic acids were subsequently extracted using Roche High Pure Viral RNA kit (Roche).

### Library preparation and sequencing

Three different library preparation kits were used; NEBNext E6070 (New England BioLabs) for total DNA, Nextera XT DNA Sample preparation kit for the enriched virion-associated DNA (Illumina), and ScriptSeq v2 (Epicentre) for total RNA and enriched virion-associated RNA. The libraries were prepared with varying input volumes according to manufacturers instructions. For shotgun DNA libraries, virion-enriched DNA libraries and RNA libraries the input volume was 43.75μl, 5μl and 9μl, respectively. All libraries were sequenced on the Illumina Hiseq 2000 platform, using paired-end reads of 100bp (PE100).

The data is available from the Short Read Archive under the BioProject ID PRJNA260349. All human reads have been deleted from the data.

### Data processing

Adapter sequences were removed and overlapping read pairs merged using AdapterRemoval [34]. Reads were mapped onto the human genome (hg19) and a reference collection of viral genomes (AC_000008.1, GU109481.1, NC_001802.1, X05015.1, the 208_p_MV_tag genome and enterovirus aRNA sequence) using bwa [35]. Quality trimming was invoked both during removal of adapters (—trimqualities and default—minquality (2) in AdapterRemoval) and mapping (-q 20 in bwa). Read pairs or merged reads with identical start and end mapping positions were considered to be of clonal origin, and only one representative for each mapping coordinate set was kept for analysis of unique reads.

For each sample, the number of unique reads mapping to each virus was recorded, with reads mapping to the human genome being discarded. Rarefaction analysis shows the diversity in a given dataset, and was performed by iteratively including sets of 1×10^5^ read sequences.

## Results

To estimate the efficiency of the enrichment procedures and the different library building techniques used for HTS, we created seven different samples consisting of human PBMCs spiked with different viruses. To mimic some of the viral diversity, the sample material contained viral genomes integrated into the human genome, intact non-enveloped virions from both DNA and RNA viruses, a DNA plasmid carrying a viral genome and aRNA particles containing a small RNA fragment originating from an enterovirus. The concentration of each virus was determined by qPCR (Table 1).

Shotgun sequencing is a simple and useful technique, but often a very expensive method when searching for low titres of viruses, as viral nucleic acids often only constitute a minute fraction compared to the host genetic material. To determine the sensitivity of DNA shotgun sequencing for viral detection, DNA shotgun libraries were prepared for all seven samples, and the individually indexed libraries were sequenced on separate lanes, producing between 143 and 191 million reads per sample (average 174 million reads). Deep sequencing was performed to ensure that viral reads were obtained from the samples containing even the lowest concentrations of viruses, making it possible to assess the level of enrichment at all concentrations.

Considering the total number of produced sequencing reads, viral genome size and quantity, the expected proportion of viral reads was calculated for the individual viruses in all samples. Table 3 lists the expected and the observed proportion of reads in each sample based on the total DNA/RNA extractions. For DNA shotgun sequencing there was generally good agreement between the observed and the expected proportion of reads. All DNA targets were detected in samples in which the expected quantity was >0, except for one of the samples with the lowest concentration of MeV plasmid (7 copies/μl).

**Table 3:** Expected and observed proportion of reads in DNA shotgun libraries.

	**Proportion of viral reads** * **(expected / observed)**			
**Sample**	**HAdV**	**HPV-18, integrated**	**HIV-1, provirus**	**MeV plasmid**
1	0.00 / 0.00	0.00 / 0.00	13 / 4.0	4.61/ 0.31
2	0.02 / 0.09	0.00 / 0.02	0.88 / 0.22	3.3/ 0.77
3	0.03 / 0.38	0.00 / 0.00	0.10 / 0.05	0.41 / 0.02
4	0.29 / 4.0	0.00 / 0.21	0.00 / 0.00	0.44 / 0.05
5	0.49 / 6.6	1.5 / 1.1	0.00 / 0.00	0.08 / 0.07
6	3.7 / 71	12 / 7.8	0.00 / 0.00	0.06 / 0.00
7	39 / 683	144 / 47	0.00 / 0.00	0.00 / 0.00

* Proportions expressed in ppm.

**Fig 2:**
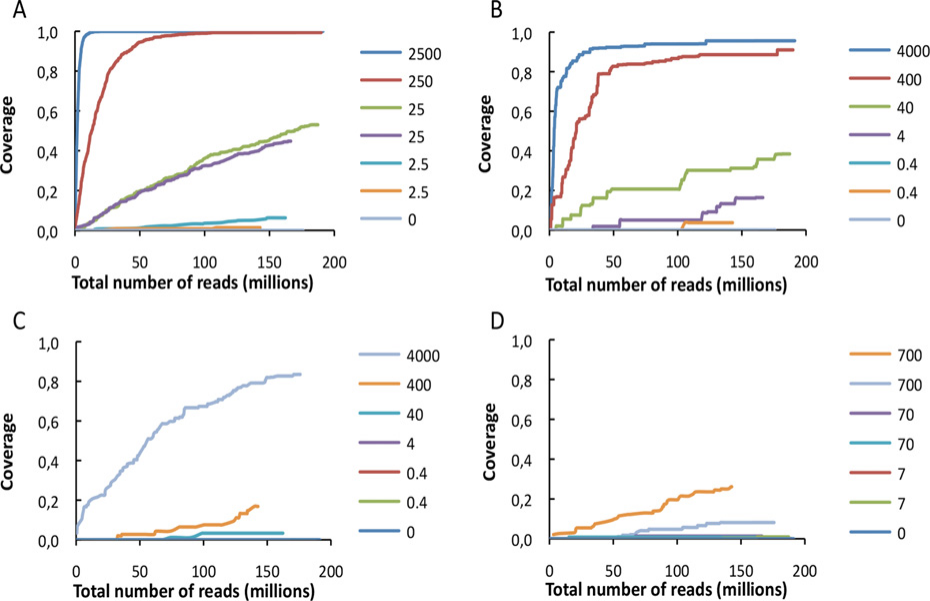
Rarefaction analysis showing the covered proportion of the (A) HAdV genome, (B) the HPV-18 genome, (C) the HI-1 genome, and (D) the MeV plasmid, as a function of the total number of sequence reads from each sample for DNA shotgun sequencing. Numbers provided for each sample are given as copies/μl in the test sample.

The two samples with the highest concentrations of HAdV (2,500 and 250 copies/μl, respectively), yielded sufficient reads to completely cover the HAdV reference genome. For the lower concentrations, the genome was only partially covered. Rarefaction analysis was employed for analysing species richness by assessing the number of new sequences found in a set interval of sequences. Results showed that the coverage approached saturation in the rarefaction analysis, hence deeper sequencing would predictably result in little additional unique viral reads (Fig 2A).

HPV-18 genomes were present in the form of HeLa cells containing 10–50 HPV-18 copies/cell of integrated HPV-18 genome [29, 30]. The two samples with the highest concentration of HeLa cells (4,000 and 400 cells/μl) resulted in >95% coverage of the integrated HPV-18 sequence (Fig 2B). The samples with lower concentrations showed that the coverage approached saturation at levels between 5% and 40% of the genome, and deeper sequencing was thus unlikely to result in additional viral reads. Only in the three samples with the highest concentration of HIV-1 proviral DNA (4,000, 400 and 40 copies/μl) we observed HIV-1 specific reads (Table 3), and the coverage of the HIV-1 genome was saturated only for the most concentrated sample (Fig 2C). The genome sizes for HIV-1 and HPV-18 are of comparable length, but with fewer copies of HIV-1 than HPV-18 present per cell in the sample material it was expected to find a proportionally lower number of sequences for the proviral HIV-1.

The coverage of the MeV-plasmid reached saturation at only 26% and 8% for the two samples with the highest concentration (700 copies/μl for both samples). For samples containing 70 or 7 copies/μl (samples 3 to 5), the coverage remained constant around 1% whereas for sample 6, having the same concentration as sample 5 (7 copies/μl), no MeV-plasmid reads were detected (Fig 2D).

For the shotgun DNA libraries, the samples were sequenced on individual lanes. Four indexes were used per sample in order to gain a higher complexity on the lane. For all other library types we used one index per sample, and the samples were sequenced in pools of three to seven samples per lane. In datasets from libraries sharing lanes, rare false positive reads were detected mapping to EV, HPV-18, HIV-1 or HAdV at frequencies ranging from 5×10^-5^ to 5×10^-7^ (see further discussion below). The libraries producing false positive reads were tested by specific qPCRs, and were in all cases found qPCR-negative.

RNA shotgun libraries were sequenced with three or four samples per lane, producing between 32 and 70 million reads per sample (53 millions on average). As the host RNA was not quantifiable, the proportion of expected viral reads could not be predicted. Under the assumption that the amount of total RNA was equal for all samples, the expected proportion of viral reads was instead calculated relative to the observed number of viral reads for the sample with the highest concentration of the given virus and normalised to the total number of reads. Table 4 lists the expected and the observed proportion of reads for each virus for each sample. In most cases the observed proportion of viral reads exceeded the predicted proportion.

**Table 4:** Expected and observed proportion of reads in RNA shotgun libraries.

	**Proportion of viral reads** * **(expected / observed)**			
**Sample**	**EV**	**aRNA**	**HIV-1**	**HPV-18**
1	0.00 / 0.48	ND	71,539^#^ / 71,539	0.00 / 0.96
2	0.12 / 1.6	ND	2,611 / 5,788	0.03 / 0.00
3	0.13 / 1.7	ND	297 / 1,058	0.03 / 0.28
4	1.7 / 16	ND	37 / 102	0.38 / 5.2
5	2.1 / 13	ND	4.6 / 18	4.6 / 54
6	17 / 109	ND	4.6 / 36	37 / 273
7	264^#^ / 264	ND	0.00 / 47	592^#^ / 592

ND: None detected.

* Proportions expressed in ppm.

^#^ Value defined by the observed.

As for the DNA shotgun sequencing, all viral targets could be detected when the sequencing depth and virus concentrations yielded a prediction of more than zero reads. However, we found a small fraction of reads mapping to HIV-1 or HPV-18, in our virus-negative controls, indicating misinterpretation of reads during parallel sequencing of multiple samples (see discussion below). HIV-1 reads exceeded the level of contaminating reads from the HIV-1 negative sample for samples containing ≥4 copies/μl. Similarly for HPV-18, RNA exceeded the background signal for concentrations ≥40 copies/μl.

**Fig 3:**
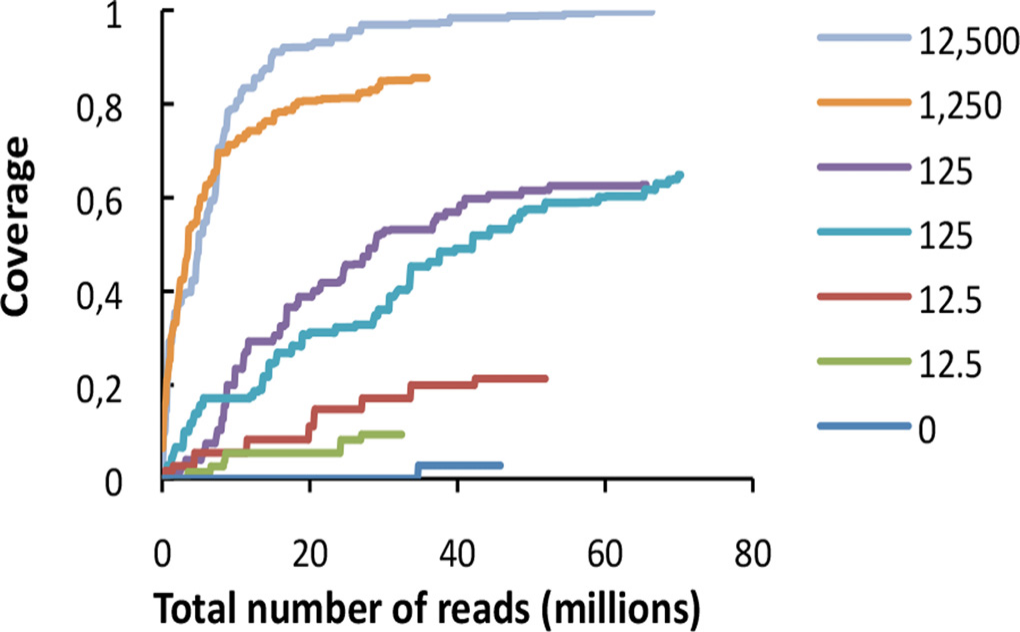
Rarefaction analysis showing the covered proportion of the EV genome as a function of the total number of sequence reads from each sample for RNA shotgun sequencing. Numbers provided for each sample are given as copies/μl in the test sample.

Coverage of the EV, aRNA, HPV-18, and HIV-1 RNA genomes was investigated. The sample with the highest concentration of EV (12,500 copies/μl) yielded sufficient reads to completely cover the EV reference genome. For the remaining samples the genome was partially covered. Results showed that the coverage approached saturation for all samples, and deeper sequencing was unlikely to result in additional unique viral reads at any of the concentrations (Fig 3).

The aRNA particles, containing a short RNA molecule, were added in equal concentrations to all samples to monitor inter-sample variation but could not be detected in any of the samples.

Expression of viral RNA was expected in the HPV-18 and HIV-1 material. RNA shotgun sequencing yielded coverage of the HPV-18 genome similar to DNA shotgun sequencing despite that five times less sample material was used.

In contrast, all HIV-1-positive fractions used for RNA shotgun sequencing showed a higher coverage of the HIV-1 genome than was the case for DNA shotgun sequencing. For RNA shotgun sequencing the lowest concentrations yielded a coverage that approached saturation at >60%, compared to less than 5% for the DNA shotgun sequencing. These results indicate that, if replicating, integrated DNA viruses or proviral retrovirus may be detected more sensitively by RNA sequencing.

We compared the sensitivity of shotgun sequencing to that of specific qPCR assays. In general, a higher input amount was required for detection by shotgun sequencing than by qPCR detection. However, in some cases a few viral reads were detected for concentrations below the detection limit of qPCR (e.g. HPV-18). Altogether the results from shotgun sequencing suggest that there may be important differences in obtained results, influenced by the type, size and location of the viral target.

As the viral nucleic acids make up only a small proportion of the total nucleic acids in most biological samples, we conducted virion enrichment on the test samples. To evaluate the effectiveness of such methods, selective removal of host genetic material by filtration and nuclease treatment was followed by extraction of DNA and RNA from remaining intact virions.

To estimate the depletion of host nucleic acids fluorometric quantification of the DNA concentration was performed for the extractions. For the DNA extracts from samples not exposed to enrichment, the DNA concentration ranged from 4.3 to 10.5 ng/μl, whereas the DNA concentration of the extracts from the samples subjected to enrichment was below 10 pg/μl. The substantially decreased DNA concentration indicates that depletion of non-virion associated genetic material was efficient.

The enrichment process targeted primarily HAdV and EV virions. However, libraries prepared from extracts of the virion-enriched samples were investigated for all the viral targets. DNA sequencing produced between 19 and 41 million reads per sample (31 million reads in average) (Table 5).

**Table 5:** Proportion of viral reads in shotgun libraries in comparison with virion-enriched libraries.

	Viral reads in shotgun libraries / viral reads in virion-enriched libraries (ppm^#^)							
	DNA libraries				RNA libraries			
Sample	HAdV	HPV-18, integrated	HIV-1, integrated	MeV plasmid	HIV-1	HPV-18	EV	aRNA
1	0 / 5.2	0 / 0	4.0 / 0.44	0.31 / 30	71,539 / 1,756	0.96 / 0	0.48 / 1.1	0 / 0
2	0.09 / 947	0.02 / 0	0.22 / 3.6	0.77 / 2.4	5,788 / 221	0 / 0	1.6 / 19	0 / 0
3	0.38 / 354	0 / 0	0.05 / 0	0.02 / 0.69	1,058 / 3,754	0.28 / 0	1.7 / 20	0 / 0
4	3.99 / 4,028	0.21 / 0	0 / 0	0.05 / 2.0	102 / 72	5.2 / 0	16 / 45	0 / 0
5	6.59 / 5,376	1.1 / 0	0 / 0	0.07 / 1.8	18 / 42	54 / 0.94	13 / 100	0 / 0
6	71 / 56,849	7.8 / 0	0 / 0	0 / 12	36 / 86	273 / 4.2	109 / 1,454	0 / 0
7	683 / 110,236	47 / 1.0	0 / 0	0 / 0.67	47 / 48	592 / 1,454	264 / 887	0 / 0

^#^ Results are presented as the proportion of the total number of unique mapping reads.

**Fig 4:**
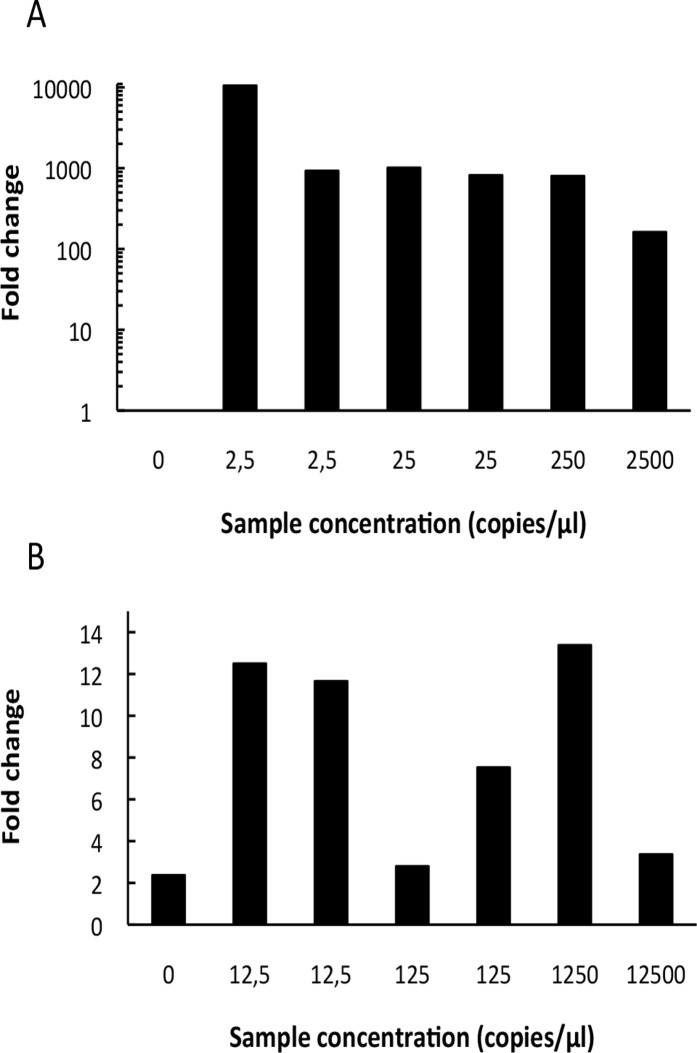
Fold increase in of the proportion of HAdV (A) or EV (B) sequences in virion-enriched libraries as compared to non-enriched shotgun sequenced libraries. Proportions are calculated as the number of viral reads relative to the total number of assigned reads.

The proportion of HAdV reads was increased for all enriched samples compared to shotgun sequencing. The virion enrichment procedure typically resulted in a 900-fold increase in the proportion of viral reads (Fig 4). For the two samples with the lowest concentration, the increase was 10,480 or 925 fold. For the sample with the highest concentration an increase of only a 161-fold was observed, suggesting that virion enrichment may be more effective at low concentrations.

For all DNA virion-enriched samples the coverage of the HAdV genome approached 100%, which was only the case for the two most concentrated samples when using DNA shotgun sequencing. Furthermore, the total amount of reads required to reach full genome coverage was lower. The increase in observed coverage was best illustrated by comparing the number of reads required to reach 50% coverage of the genome (Table 6).

**Table 6:** Sequencing required to reach threshold coverage of viral target genomes.

	**DNA libraries (HAdV)**			**RNA libraries (EV)**		
	**Copy no.**	**Reads required for 50% coverage (x10^6^)**		**Copy no.**	**Reads required for 50% coverage (x10^6^)**	
**Sample**	**per μl sample**	**Virion-enriched**	**Shotgun-sequenced**	**per μl sample**	**Virion-enriched**	**Shotgun-sequenced**
1	0	NA	NA	0	>29	>45.6
2	2.5	1.3	>143	12.5	>31	>51.2
3	2.5	2.3	>162	12.5	>24	>32.4
4	25	0.2	>166	125	3.0	29.1
5	25	0.2	164	125	0.9	42.1
6	250	<0.1	12.7	1,250	<0.1	3.6
7	2,500	<0.1	1.7	12,500	0.4	5

Even for the lowest concentrations of 2.5 copies/μl as little as 1.3 and 2.3 million reads resulted in 50% coverage of the 36 kb genome, suggesting that an even lower concentration of HAdV could have been detected by virion enrichment.

As expected, the ratio of human reads decreased in enriched libraries (28% on average) compared to shotgun libraries (88% on average). Furthermore, the number of reads and genome coverage was drastically reduced for HIV-1 and HPV-18 DNA in libraries from virion-enriched fractions. Likewise generally only a few MeV plasmid reads were obtained in these datasets. This was the case for all samples except for sample 1, which had an input of 700 copies/μl, and yielded a higher proportion of MeV plasmid reads for enriched DNA libraries compared to DNA shotgun libraries, something we currently cant explain.

Libraries prepared from the virion-enriched RNA yielded between 14.5 and 44.6 million reads (26.3 million on average). EV was the only virus for which enrichment could be expected after this treatment, and to some extent HIV-1, as the 8E5 cell line is capable of producing immature viral particles. Again, the proportion of EV viral reads was increased for the enriched samples compared to the shotgun samples. A 2 to 13 fold enrichment was observed in the proportion of EV sequences between shotgun and virion-enriched RNA libraries. The fold change fluctuated and no clear trend was observed between the different viral concentrations. (Table 5, Fig 4B). As for the DNA virion enrichment, an increase in genome coverage was observed by RNA virion enrichment. At a concentration of 125 copies/μl between 0.9 and 3 million reads were required to reach a coverage of 50% of the genome (Table 6). As for the DNA libraries, the ratio of HPV-18 reads was lower for virion-enriched samples than for shotgun samples. Furthermore the amount of human reads decreased from an average of 92% in shotgun libraries to an average of 69% in virion-enriched libraries. The aRNA could not be detected in the samples from either shotgun RNA or virion enriched RNA libraries.

We have confirmed that shotgun sequencing produces the expected number of reads for low concentrations of virus, but that the type of virus may affect the detection limit, which is important for virus discovery. Our results show that virion enrichment may provide approximately one or three orders of magnitude for varying dilutions of viral target RNA or DNA, respectively. Importantly, our results were inconclusive with regards to detection level, supporting neither enrichment nor shotgun as the most sensitive approach. The enrichment procedure offers a more cost-effective sequencing, which also requires more handling of the individual sample.

## Discussion

Viruses cause a variety of different diseases, ranging from completely asymptomatic, to common colds, and life-threatening illnesses including cancer in humans and animals [1, 4, 18]. The etiology of many febrile diseases, chronic conditions and cancers remain unknown, and the search for novel viruses continues to be of great importance [7]. Searching for viruses in complex biological samples has proven challenging, due to low viral concentration in combination with great genetic diversity. High-throughput sequencing, sometimes in combination with upstream enrichment, has been used to identify viruses in a variety of samples [25, 36–42], but the sensitivity or effectiveness of these methods is rarely assessed.

In the present study, we mimicked human sample conditions by spiking human background material with different types of viruses to determine the effect of virion enrichment and the sensitivity of high-throughput sequencing. Four different approaches were compared; shotgun DNA or RNA sequencing as well as virion-enriched DNA and RNA sequencing. Seven different sample compositions were investigated. Virion enrichment was achieved by physical removal of host cells and cellular debris via centrifugation and filtering followed by enzymatic removal of host genetic material.

We observed 7 cases in which reads mapped to viruses (HPV-18, HAdV5, HIV-1, EV and MeV) that were not added to the sample. In all cases individually indexed libraries shared sequencing lanes with other libraries containing high quantities of the same virus. The relevant libraries were all negative for viral sequences when tested in sensitive target-specific qPCR, suggesting that laboratory inter-sample contamination was unlikely. Furthermore, extensive shotgun sequencing of samples not sharing lanes (>1.4×10^8^ reads) showed no indications of contamination. Together this argues that the reads appear as an artefact of misreading of clusters or indexes during sequencing of several samples per lane. It has been shown that low numbers of contaminating reads can be extremely difficult to avoid. In a carefully controlled study up to 5000 parts per million (ppm) of all indexed reads were misinterpreted during Illumina sequencing, contributed also by carry-over of indexes during laboratory handling, or manufacturing of oligonucleotides [43]. Here, the observed level of artefact sequences range between 0.48–48 ppm (Table 5), which is probably to be expected when processing samples containing high virus concentrations together with negative controls. In this study all negative samples producing viral reads, were sequenced together with the sample with the highest concentration of those particular viruses. However, when using these sequencing methods for viral discovery, where high viral titres are the exception, the risk of contaminating reads may be negligible.

In our experiment, the proportion of reads in the negative controls defines the threshold proportion for considering our test samples truly positive. In the case of HIV-1 reads, we cannot exclude that reads in sample 5 and 6 (each with 0.4 copies/μl) also stem from sequencing artefacts. For virion-enriched HAdV DNA the background level is an order of magnitude lower than the signal in the expected positive samples. For shotgun sequenced HPV RNA the background was 0.96 ppm. In all cases the background sequences had no effect on our conclusions.

We used extensive shotgun sequencing to provide a foundation for evaluating the efficiency of virion enrichment at all viral concentrations. Shotgun sequencing results confirmed that viral reads could be detected at even the lowest concentration with extensive sequencing. With viral concentrations of 2.5 copies/μl, obtaining a 50% coverage of HAdV DNA required more than 143×10^6^ reads. Previous studies have reported high sensitivity by HTS [44–47]. Malboeuf *et al*. obtained 96–100% coverage of their viral targets with 5 million total reads using 100 viral copies/reaction [45]. Another study reported detection of viral RNA diluted a million times compared to human RNA [47]. However, in these studies, target amplification was performed prior to preparation of the libraries [44–46] or extracted host material was spiked with viral extract [47], which can explain the different level of detection.

This study confirms the theoretical expectation that any virus, DNA or RNA, can be detected in a complex sample, as long as the depth of sequencing is sufficient. This finding provides a necessary foundation for evaluating the effectiveness of virion enrichment as well as other enrichment methods.

In our study, different types of viruses were spiked into the samples at varying concentrations prior to extraction and no amplification was performed prior to preparation of the libraries. Using this approach, we detected HIV-1 provirus reads at concentrations down to 40 copies/μl starting material, and HPV-18 at a lower level corresponding to the 10–50 copies per HeLa cells [29, 30] (0.4–4 cells/μl each with 10–50 copies/cell). With shotgun RNA sequencing we detected EV down to a concentration of 125 copies/μl starting material. These findings indicate that the employed DNA and RNA HTS techniques in this study may be equally sensitive, and only less sensitive than the level reported with target pre-amplification [44–46]

The coverage of the MeV plasmid was low in all samples upon DNA shotgun sequencing. Even for the highest concentrations of MeV plasmid (700 copies/μl) the coverage reached merely 26%, indicating that plasmid DNA may be difficult to detect by DNA shotgun sequencing. Preliminary experiments have shown that plasmids of a similar size and quality may be resistant to fragmentation by sonication (using a Bioruptor) performed prior to building the DNA library. We speculate that this resistance is caused by super-coiling which could explain the low number of reads detected by DNA shotgun sequencing. Other methods targeting circular DNA, such as φ29-mediated amplification [48, 49], have proven highly efficient in selective amplification of circular DNA virus genomes [50].

Generally, we detected fewer viral reads in RNA than DNA sequencing in both shotgun and virion-enriched libraries. There are several possible explanations for this. In any sample the concentration of viral RNA would expectedly be very low. Before preparation of RNA shotgun libraries the extracted nucleic acids were digested with DNase, and thus underwent two purification steps prior to cDNA synthesis, using silica columns, both of which lead to a loss of material. Likewise, the fraction used for virion-enriched RNA libraries was nuclease-digested prior to extraction, during which some loss of RNA may occur. Subsequent DNase treatment prior to RNA library preparation was initially attempted, but omitted as the resulting DNA concentrations were too low to support library preparation.

Obtaining a low number of RNA sequences is not unusual when using RNA shotgun sequencing. In one study only 14 novel arenavirus reads were detected in a sample prepared from pools of fractions with concentrations between 16,600 and 2.3×10^6^ viral RNA copies per ml of extract [25]. The low quantities of reads emphasize the need for deep sequencing and/or implementation of target enrichment procedures.

Two different kits were used for preparation of the DNA libraries. For low quantities of DNA upon virion-enriched, we used Nextera XT optimized for small amounts of DNA. For total DNA libraries, the NEBNext E6070 was selected for its ability to include large amounts of DNA, increasing library complexity and the possibility to obtain viral reads. All RNA libraries were prepared using ScriptSeq v2. It was difficult to estimate the potential impact of the different library kits. A place to look for differences could be the fragment lengths of the prepared libraries. The fragment lengths within the RNA libraries were approximately the same fluctuating between 150 and 500 bp with an average length of 350 bp. For the shotgun DNA libraries the fragment length within the libraries varied between 150 bp and 1000 bp. The same applied to the virion-enriched libraries; however, they tended to peak at around 200 bp, where the shotgun library fragment lengths were evenly distributed and thus in general longer than the virion-enriched libraries. This could indicate a small difference in the performance of the kits, however we assume this difference was negligible. Even though the lengths varied between the two types of DNA libraries, the applied kits provided the optimal conditions for viral discovery and thereby provided the best foundation for assessing the effect of virion enrichment.

In the present study, sample concentrations exceeding 250 viral copies/μl resulted in a relatively high coverage with shotgun DNA and RNA sequencing, indicating that relatively high viral concentrations were required to obtain full coverage (Fig 2B). This clearly illustrates a challenge for detection of novel viruses, as *de novo* assembly may prove difficult when suboptimal coverage is obtained and subsequent targeted molecular methods are often required [17, 25, 36, 37].

We added equal amounts of aRNA (encoding 263 bp 5’UTR of enterovirus) to all samples as an inter-sample variation control. However, aRNA was not detected in any of the shotgun or enriched RNA samples (Table 5). The added concentration is readily detectable by qPCR when extracted alone. When preparing libraries using the ScriptSeq kit, RNA is initially primed by random hexamers during cDNA synthesis. The random hexamers are less likely to hybridize to the target RNA in the presence of high levels of competing RNA. We therefore speculate that the RT reaction in preparation of complex libraries may introduce bias against very short stretches of RNA.

It is evident that target enrichment can reduce the number of sequence reads required to obtain a certain coverage. It is less clear if target enrichment may actually result in improved sensitivity via a lower detection limit. We compared detection of virus in virion-enriched DNA and RNA libraries with shotgun sequenced libraries (Table 5). The results showed that enrichment of virion-associated DNA was successful and typically increased viral target sequences by three orders of magnitude. For the virion-associated RNA, viral target reads was increased up to 13 fold. The viral concentration seemed to have limited effect on the degree of DNA or RNA enrichment (Fig 4). Hall et al., 2013 [27] has previously characterized the effects of virion-enrichment on viral targets, using an enrichment approach similar to ours, with centrifugation, filtration and nuclease treatments. They showed that HAdV copynumbers were not affected by their enrichment treatments but that EV copynumbers were reduced 100 fold by enrichment procedures. The sequencing of EV yielded a 20-fold increase compared to samples that were not subjected to enrichment procedures, which is similar to what we detected. The 100 fold decrease in EV copynumber, could explain the lower increase in viral sequences observed for EV compared to HAdV in this study.

This study shows that viral detection is possible without the use of random amplification prior to library preparation by using virion enrichment. This is advantageous, as bias may be introduced during such pre-amplification. When searching for viral targets in biological samples several approaches exist. This study also emphasizes the importance of choosing the appropriate method, which will depend greatly on the titre and types of viruses present in the sample. Our results indicate that enrichment and shotgun methods are equally sensitive, as viral reads were detected at all concentrations for both approaches.

Deciding the sufficient shotgun sequencing depth is difficult when the proportion of the viral component is unknown. Viral titres may vary, and may be high in acutely infected patients, but more often, viruses will be present in rather low titres. When using shotgun sequencing the major expense is currently the sequencing reagents, whereas virion enrichment is more labour-intensive and has additional reagent costs. Shotgun sequencing can be cost-prohibitive, but given the continually decreasing prices for HTS [24], it may become the most appealing future virus discovery approach.

For RNA, the enrichment was no more than 2–13 fold. Our results support that for some viral targets (e.g. EV), a proportion may be lost during laboratory handling. This suggests that other target enrichment procedures should be considered at potentially low concentrations of target viruses. Alternative viral enrichment techniques include hybridization capture [15, 22, 51], depletion by capture of host material [23] or rolling circle amplification of viral targets [48].

Our study provides an estimate of the effectiveness of virion enrichment, as viral targets were detected at the lowest concentrations possible using shotgun DNA and RNA sequencing. Other enrichment methods could be applied to the samples produced in this study in order to estimate their degree of enrichment. Despite virion-enrichment reducing the required sequencing effort, it is not evident from our data that a lower detection level is actually achieved.
